# The utility of electronic frailty index in cancer patients undergoing chemotherapy

**DOI:** 10.1038/s41416-026-03389-y

**Published:** 2026-04-10

**Authors:** Agnieszka Michael, Jennie Huynh, Katie Sutton, Man-Chie Chow, Elizabeth Ford, Janine Mansi, Peter Selby, Simon Skene, Jo Armes

**Affiliations:** 1 University of Surrey, Guildford, United Kingdom; 2 University of Edinburgh, Usher Institute, Edinburgh, United Kingdom; 3 University of Surrey, School of Health Sciences, Kate Granger Building, Guildford, United Kingdom; 4 Royal Surrey NHS Foundation Trust, Oncology, Guildford, United Kingdom; 5 Brighton and Sussex Medical School, Division of Public Health and Primary Care, Brighton, United Kingdom; 6 Guy’s and St Thomas’ NHS Foundation Trust, London, United Kingdom; 7 St James’ University Hospital, Cancer Research Building, Leeds, United Kingdom; 8 Clinical Research Building, Guildford, United Kingdom

**Keywords:** Breast cancer, Non-small-cell lung cancer, Colon cancer

## Abstract

**Background:**

Frail patients with cancer (Ca) have worse survival. Current methods of assessment of fitness (performance status) for cancer treatment, such as chemotherapy, are time -consuming and often not used by practicing oncologists. The electronic frailty index (SCARF) is derived from a cumulative deficit frailty model and provides a measure of frailty alongside pre-existing conditions. We used this methodology to investigate whether it can predict outcomes of chemotherapy in patients with Ca.

**Methods:**

The study conducted data analysis of Ca patients treated with chemotherapy in England, years 2015–2018; stage II–III breast Ca, stage III colon Ca and stage IIIB–IV non-small-cell lung Ca. The data was linked with hospital admissions to calculate 30-day chemotherapy mortality, overall survival and SCARF.

**Results:**

The SCARF was calculated for 78,799 patients. The risk of dying within 30 days of chemotherapy in severely frail patients with colorectal cancer ≥70 y.o. was twice that of the <70 y.o. (OR 2.04 −95% CI 1.58–2.64, mild frailty 1.07–95% CI 0.78–1.45); and 6 times higher in breast cancer (OR 5.73–95% CI 2.66–12.32, mild frailty OR 1.45 95% CI 0.78–2.71).

**Conclusion:**

The SCARF index predicts poor outcomes from SACT, particularly in breast and colon cancer, and it requires further evaluation.

## Introduction

Older and frail patients with cancer have lower curative treatment rates than patients who are younger. There is evidence that older patients who are often fit, receive less aggressive treatment and as a result have worse survival [1, 2]. We also know that many older and frail patients often do not tolerate systemic chemotherapy well and are frequently harmed as a result of treatment [3]. Current methods of assessment of fitness for chemotherapy, such as performance status (PS) are often inconsistently applied and fail to capture the complexity of age related vulnerabilities [3, 4]. There is evidence that the use of geriatric assessment can help with the management of treatment toxicity; however, data on outcomes such as quality of life and survival is less clear [5]. The complexity of geriatric assessment, lack of training and time pressures in busy clinics, as well as difficulties that clinicians face when deciding on the correct treatment for frail patients, mean that better solutions are needed [4]. Developments in the digital field and electronic sources of information give new opportunities and offer solutions that can be accessed at the point of referral from general practitioners and have a potential to influence daily clinical practice.

Frailty is a condition of increased vulnerability to major changes in health often because of seemingly small problems, such as infection or new medication. It is common in older age, affecting around 10% of people aged over 65 and up to 42% of older cancer patients in UK [6]. People with frailty are at increased risk of falls, disability, loneliness, hospitalisation, and care home admission. These problems can reduce quality of life and are costly for health and social care [6].

The International Society of Geriatric Oncology (SIOG) recommends that practitioners perform a comprehensive geriatric assessment (CGA) to profile patient frailty levels both to optimise therapeutic decisions and to help estimate life expectancy [7]. Full CGA requires expertise and time, and such assessments are not available in the majority of the centres in UK. Simplified “screening” tools such as G8 and the Vulnerable Elders Survey (VES-13) have been proposed to help oncologists to identify patients who need a full geriatric assessment and a referral to a geriatrician but uptake in the UK remains low [7].

In the absence of structured geriatric assessment, treatment decisions are typically based on clinician judgement, patient preferecne, comorbidities and PS scores such as the Eastern Cooperative Oncology Group (ECOG) (adopted by WHO) or Karnofsky scale [8]. In practice, however, there is evidence that the ECOG scale may not be the best way to assess patients for chemotherapy. These scales demonstrated limited reliability and inter-rater consistency and may exclude patients who could still benefit from the treatment [9–11].

A UK initiative – the electronic frailty index (eFI) – has been derived from a cumulative deficit frailty model and provides a measure of frailty alongside pre-existing conditions in UK primary care; frailty and multimorbidity are related in older adults [12]. Clegg et al. analysed data from 931,541 patients using 36 deficits (Table 1) that are common in the general population, such as arthritis, cardiac abnormalities, history of falls, cognitive problems, polypharmacy and a number of other comorbidities [13]. Based on these 36 clinical deficits, they developed and validated an electronic frailty index (eFI) that is automatically populated from routinely collected data contained within primary health care records. Patients were classified into the following groups: no frailty, mild, moderate or severe frailty. One-year adjusted hazard ratios (HRs) for mortality were 1.92 (95% CI 1.81–2.04) for mild frailty, 3.10 (95% CI 2.91–3.31) for moderate frailty and 4.52 (95% CI 4.16–4.91) for severe frailty. Mild to severe frailty was also associated with the risk of hospital admission as well as the likelihood of admission to a nursing home within one year.

**Table 1:** List of 36 deficits contained in the eFI

List of 36 deficits contained in electronic frailty index	
Activity limitation	Skin ulcer
Memory and cognitive problems	Food Problems
Anaemia and haematinic deficiency	Sleep disturbance
Mobility and transfer problems	Fragility fracture
Arthritis	Social vulnerability
Osteoporosis	Hearing impairment
Atrial fibrillation	Thyroid disease
Parkinsonism and tremor	Heart failure
Cerebrovascular disease	Urinary incontinence
Peptic ulcer	Heart valve disease
Chronic kidney disease	Urinary system disease
Peripheral vascular disease	Housebound
Diabetes	Visual impairment
Polypharmacy	Hypertension
Dizziness	Weight loss and anorexia
Requirement for care	Hypotension/syncope
Dyspnoea	Ischemic heart disease
Respiratory disease	
Falls	

A similar index called SCARF (Secondary Care Administrative Records Frailty index) has been developed based on hospital records and successfully used in breast cancer and colorectal cancer studies [14, 15]. It uses the same fields as eFI except for polypharmacy. Hospital-derived index may be a better tool for oncology patients treated in hospital setting as this data is more likely to be available and easy to access. The information on SCARF would allow the clinician to discuss potential risks of chemotherapy tailored to the individual patients.

With growing pressures on cancer services, the digitisation of healthcare provides an opportunity to integrate frailty assessment into routine practice. The development of the SCARF and eFI indices in the UK offers a foundation for evaluating whether such tools can predict chemotherapy-related adverse outcomes. We conducted a mixed-method study based on retrospective chemotherapy records in the UK to evaluate SCARF as a tool to predict 30-day mortality and hospital admissions.

### Aims and objectives

The overall aim of the study was to investigate whether SCARF is predictive of adverse outcomes of chemotherapy in frail patients with cancer stratified for age in view of higher incidence of frailty in older patients (≥70 y.o. and ≤69 y.o.). The adverse effects of chemotherapy were defined as 30-day mortality from chemotherapy and hospital admissions during chemotherapy. The 30-day period was defined as 30 days from day 1 of the chemotherapy cycle, immediately prior to death or if chemotherapy was continuous, as 30 days from the date of the last chemotherapy prescription [16].

The secondary objectives were:

- A comparison of SCARF with ECOG PS as predictors of adverse outcomes of chemotherapy.
- The predictive value of SCARF in overall survival (OS).

### Study design

The study was designed as two work-packages: work-package 1 (WP1) comprised data analysis of retrospective data on cancer patients treated with chemotherapy obtained from the National Cancer Registration Analysis Service (NCRAS) and Systemic Chemotherapy Dataset (SACT). Work-package 2 (WP2) was a qualitative study that explored patients, carers’ and clinicians’ attitudes to using an automated frailty score that can inform the decision-making process [17]. Patients participating in the qualitative study provided INFORMED consent.

The data analysis project included historical SACT records obtained from NCRAS from 2015 to 2018

Patients included in the analysis had stage II or III breast cancer, stage III colon cancer or stage IIIB–IV NSCLC as described below in the cohort characteristics.

Data from SACT were linked with hospital admissions statistics to calculate 30-day chemotherapy mortality and overall survival for this cohort and hospital admissions during this time (starting day 1 cycle 1 to six months after day 1 cycle 1-last cycle, plus 30 days). The follow up for each patient was six months (starting day 1 cycle 1 to six months after day 1 cycle 1).

### Cohort

The National Cancer Registration Analysis Service is the population-based cancer registry for England. It collects, quality assures and analyses data on all people living in England who are diagnosed with malignant and pre-malignant neoplasms in the National Health Services (NHS) [18]. From the cancer registry closedown table 2018, all patients with a first primary stage II or stage III breast cancer (International statistical classification of diseases and related health problems version 10 (ICD-10) code C50), stage III colorectal cancer (ICD-10 C18-C20) or stage IIIB–IV non-small-cell lung cancer (ICD-10 C33-C34, and excluding small cell lung cancer, ICD-O-3 morphology codes 8002, 8041, 8042, 8043, 8044 and 8045) aged between 18 and 125 with a known gender, NHS number and lower super output area (LSOA) code between 2015 and 2018 were identified (*n* = 196,822).

### Patient demographics

Information on patients’ age, gender and ethnicity information was taken from the cancer registration data. Socioeconomic deprivation was derived from the quintile distribution of the index of multiple deprivation (IMD) 2019 from the Office for National Statistics (ONS) and allocated based on the patient’s LSOA at time of diagnosis, with 1 representing the most deprived and 5 representing the least deprived areas.

### Systemic anti-cancer therapy data

All patients were linked to the Systemic Anti-Cancer Therapy (SACT) database, a national dataset which records data on all SACT activity provided by the NHS in England [19]. Linkage was established using the patient’s NHS number and the 3-character ICD-10 codes. Linkage was established for 78,841 tumours. We excluded treatments with any benchmark group drugs, defined as zoledronic acid, pamidronate, not chemo, hormones, denosumab, or missing information. A first SACT regimen was defined as the first treatment dose starting between 31 days before and up to 183 days after the diagnosis date.

### Linkage to HES

The cohort was also linked to the Hospital Episode Statistics (HES) data containing details about NHS admissions, outpatient appointments and A and E attendances at NHS hospitals in England, using matching algorithms on NHS number, date of birth, gender and post code at diagnosis.

SCARF index was constructed as described in ref. [15]. Due to nature of dataset available from by Public Health England based on Hospital Episode Statistics (HES) SCARF does not contain information about polypharmacy and it therefore differs from the original eFI as it contains 35 fields instead of 36 as published by Clegg et al. [13].

The SCARF index was constructed using HES admitted patient care (APC) data. The diagnosis fields were searched for using ICD10-codes representing frailty as defined by Jauhari et al. from two years before up to the date of diagnosis. Patients were categorised into different deficit groups based on the number of deficits present: fit = 0–1 deficit (index: 0–0.05), mild frailty = 2–3 deficits (index: 0.06–0.11), moderate frailty = 4–5 deficits (index: 0.12–0.18) and severe frailty = 6 or more deficits (index: ≥0.19) [15].

### Charlson comorbidity index

The Charlson comorbidity index was based on diagnostic codes during HES admissions from 27 to three months prior to diagnosis and weighted according to Quan [20].

### Performance status (PS)

Eastern Cooperative Oncology Group (ECOG) PS at time of diagnosis was used [21].

### Emergency admissions

Emergency admissions for cancer were identified from the HES APC dataset when they occurred from the first day of the first cycle of SACT up to 183 days after and matched on the 3-character ICD-10 codes in the diagnosis fields.

### Survival

Vital status information was taken from ONS with follow up to 5th January 2020. Survival time was calculated from the date of diagnosis or the date of the first SACT event till death. Forty-two patients were excluded from the cohort due to date inconsistencies, resulting in a final cohort of *n* = 78,799.

### 30-day mortality and hospitalisation

30-day mortality was defined as death occurring 30 days after the last event of the first SACT regimen. If the last cycle or administration was administered via oral drugs, 28 days were added to the last event date to allow for completion of treatment [4]. Hospital admissions were included where they occurred during chemotherapy and up to 30 days after the last event of the first SACT -total of 6 months from start of chemotherapy.

### Statistical analysis

Differences in SCARF and Charlson index and (ECOG PS) were compared according to age (≥70 years and ≤69 years), emergency admissions, 30-day mortality and by cancer site, and tested using the χ^2^ distribution. Logistic regression was performed to calculate the odds ratios for death within 30 days after SACT according to age ≥70 years and ≤69 years of age at diagnosis, and in a multivariable model adjusted for gender, ethnicity, socioeconomic deprivation, and sequentially for SCARF, Charlson index and PS. Logistic regression models were run separately according to the cancer site. In addition, separate logistic regression models were run for patients undergoing immunotherapy. Overall survival was calculated using Kaplan–Meier estimates. Cox proportional hazards modelling was used to estimate the hazard of death according to age (≥70 years and ≤69 years of age at diagnosis) and in a multivariable model adjusted for gender, ethnicity, socioeconomic deprivation, and sequentially for SCARF, Charlson index and ECOG PS, separately for the different cancer sites and for immunotherapy. Cox proportional hazards assumptions were checked.

## Results

We examined records of 78,799 patients: 17,951 with colorectal cancer; 22,052 with lung cancer; and 38,796 with breast cancer and calculated the SCARF index for all these patients. 20,388 (26%) of the patients were ≥70 years and 58,411(74%) were ≤69 years. All patients in the colorectal cohort had stage 3 cancer, in the breast cancer cohort: 74% (28,727) had stage 2 and 26% (10,069)-stage 3 cancer, and in the lung cancer cohort: 80% (17,630) had stage 4 cancer and 20% (4422) had stage 3.

When examining the colorectal, lung and breast cancer patients together, 69% were classified as fit (see Table 2), 19% were classified as having mild frailty, 7.7% as moderate frailty and 3.6% (2837) as severe frailty.

**Table 2:** The summary of Charlson score, SCARF index and performance status as well as chemotherapy adverse outcomes: 30-day chemotherapy mortality and emergency admissions within 6 months

	Colorectal	Lung	Breast	Total
Charlson score				
0	15,809	18,032	35,989	69,830
1–2	1864	3332	2550	7746
3+	278	688	257	1223
SCARF				
Fit	10,268	9946	34,349	54,563
Mild frailty	4997	7227	3071	15,295
Moderate frailty	1900	3207	997	6104
Severe frailty	786	1672	379	2837
Performance status				
0	7952	4550	25,798	38,300
1	6493	11,281	6468	24,242
2	453	2932	330	3715
3-4	24	306	35	365
Missing	3029	2985	6167	12,181
30-day mortality				
*N*	17,711	19,035	38,697	75,443
Y	240	3017	99	3356
Emergency admission within 6 months				
*N*	17,068	18,115	36,568	71,751
Y	883	3937	2228	7048

Whilst most patients were assessed as having an ECOG PS of 0 (48%) or 1 (30%), a significant proportion (15%) did not have ECOG PS documented.

4.2% of patients treated with chemotherapy died within 30 days of chemotherapy and 8.9% had an emergency admission that was attributed to chemotherapy toxicity (during 6 months from the 1st cycle of chemotherapy and 30 days after the last cycle)

### The overall survival, 30-day mortality and hospital admissions in patients with colorectal cancer treated with adjuvant chemotherapy ≥70 years old compared to patients ≤69 years old

**Fig. 1:**
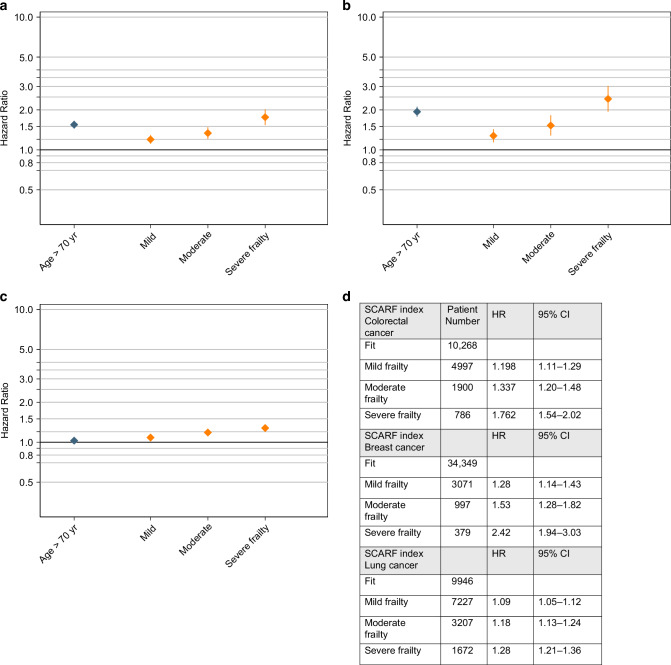
Figure representing the risk of poor overall survival for patients ≥70 y.o. comparing to ≤69 y.o. according to the SCARF index. Figure **A** shows hazard ratio for patients with colorectal cancer, Figure **B** breast cancer and Figure **C** lung cancer. Figure **D** shows the numerical values for HR and 95% CI values for OS.

The analysis of OS in patients with colorectal cancer treated with adjuvant chemotherapy, revealed that patients ≥70 years have a higher risk of dying with a HR of 1.64 (95% CI 1.53–1.75). Patients who have a SCARF indicating mild frailty had a HR of 1.2 (95% CI 1.11–1.29); those with moderate SCARF −1.34 (95% CI 1.21–1.48) and severe −1.76 (Table 3) (Fig. 1A). PS was also predictive of poor OS in patients ≥70 years old, however, a substantial proportion of data was missing (*n* = 3,029,17%). The Charlson score was not predictive and was outperformed by the SCARF index.

**Table 3:** The SCARF index, ECOG PS and risk of poor OS in patients treated with adjuvant chemotherapy for cancer patients ≥70 years compared those ≤69 years

Colorectal cancer patients										
SCARF index	OR	95% CI		Number of patients	Performance status	OR	95% CI			Number of patients
Fit				10,268	0					7952
Mild frailty	1.07	0.78	1.45	4997	1	1.33	0.99 1.79			6493
Moderate frailty	1.60	1.10	2.33	1900	2	2.59	1.47 4.58			453
Severe frailty	2.13	1.34	3.39	786	3+	10.60	3.06 36.71			24
					Missing	1.23	0.83 1.81			3029
Breast cancer patients										
SCARF index	OR	95%	CI	Number of patients	Performance status	OR	95%		CI	Number of patients
Fit				34,349	0					25,798
Mild frailty	1.45	0.78	2.71	3071	1	3.50	2.11	5.80		6468
Moderate frailty	3.50	1.82	6.75	997	2	17.7	8.69	36.28		330
Severe frailty	5.73	2.66	12.3	379	3+	82.9	28.0	245		35
					Missing	2.38	1.29	4.37		6167
Lung cancer patients										
SCARF index	OR	95%	CI	Number of patients	Performance status	OR	95%	CI		Number of patients
Fit				9946	0					4550
Mild frailty	1.17	1.07	1.80	7227	1	1.88	1.65	2.14		11,281
Moderate frailty	1.28	1.15	1.44	3207	2	4.29	3.71	4.95		2932
Severe frailty	1.48	1.28	1.71	1672	3+	10.37	8.03	13.38		306
					Missing	2.45	2.10	2.86		2985

Of the 17,711 patients with colorectal cancer, 240 (1%) died within 30 days of chemotherapy regardless of age.

**Fig. 2:**
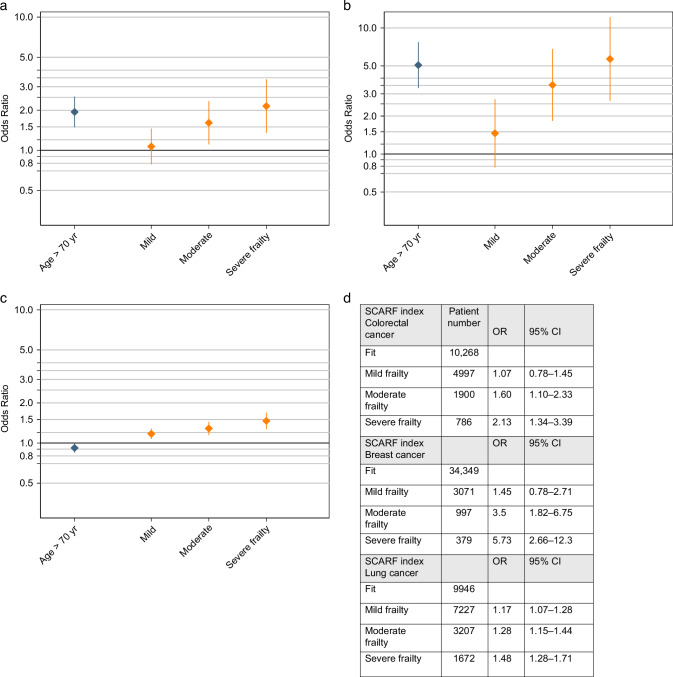
Figure representing the risk of dying within 30-days of chemotherapy for patients with colorectal, lung and breast cancer according to the SCARF index. **A** shows OR for colorectal cancer, **B** breast cancer and **C** lung cancer. Figure **D** shows the numerical values for OR and 95% CI.

The risk of dying within 30 days of chemotherapy in patients ≥70 years was twice that of those ≤69 years old (OR 2.13; CI 1.34–3.39). The SCARF index closely reflected the risk for those with mild, moderate and severe frailty with patients who had a mild frailty index having a similar risk of dying to fit patients: OR 1.07 (CI 0.78–1.45), those with moderate −1.6 (CI 1.1–2.33) and those with severe frailty −2.13 (CI 1.34–3.39) (Fig. 2A).

Hospital admissions statistics during chemotherapy showed that admissions for patients ≥70 years old did not differ from the younger patients and the OR was 0.88 for ≥70 years old (95% CI 0.77–1.02). Patients who were frail, had a higher risk of admission than those who were not frail (OR for severe frailty 1.26 95% CI 0.91–1.73). Similarly poor ECOG PS was linked with a higher risk of admission (OR for ECOG PS 3 3.05 95% CI 0.85–10.32) however it was not statistically significant. The predictive power of Charlson score was lower (OR for 3+ score-1.45 95% CI 1.45–3.30). An in-depth analysis of causes of admissions has not been done.

### The overall survival, 30-day mortality and hospital admissions in patients with breast cancer treated with adjuvant chemotherapy, ≥70 years old compared to patients ≤69 years old

Patients with breast cancer who were ≥70 years old treated with adjuvant chemotherapy had a much worse overall survival that those ≤69 years (HR 2.24; 95% CI 2.06–2.44). The risk of dying for patients with mild frailty was 1.28 (95% CI 1.14–1.43), moderate frailty HR of 1.53 (95% CI 1.28–1.82), and severe frailty HR of 2.42 (95% CI 1.94–3.03) (Table 3) (Fig. 1B). Poor ECOG PS was also predictive of poor OS but a high proportion of data were missing (15%).

Of the 38,796 patients with breast cancer, 99 (0.2%) patients died within 30 days chemotherapy. The risk of dying within 30 days of chemotherapy in patients with breast cancer who were ≥70 years was much higher than those of the ≤69 years with an OR of 5.73(95% CI 2.66–12.3). SCARF index closely reflected the increasing risk of death in patients with increasing frailty (Fig. 2B).

Hospital admissions statistics during chemotherapy showed that admissions for patients ≥70 years old did not differ from the younger patients; the OR for at least one admission within 6 months of the first day of chemotherapy treatment was 0.74 for ≥70 years (95% CI 0. 64–0.86). Patients who were frail, had a higher risk of admission that those who were not frail (OR for severe SCARF 1.46 95% CI 1.00–2.15). Similarly, poor ECOG PS was linked with a higher risk of admission (OR for ECOG PS 3 + 0.91 95% CI 0.70–5.70).

### The overall survival, 30-day mortality and hospital admissions in lung cancer patients treated with first-line chemotherapy, ≥70 years old compared to patients ≤69 years old

The OS for patients with lung cancer who were ≥70 years and treated with first-line chemotherapy did not differ from patients who were ≤69 years old (HR 1.09; 95% CI 1.05–1.12). Higher SCARF index showed that with increasing frailty, overall survival was worse; however, the difference between the mild, moderate and severe was small (Table 3) (Fig. 1C).

Overall, patients treated for the lung cancer with first-line chemotherapy had a much higher risk of dying. Of 19,035 patients, 3017 (14%) died within 30 days of chemotherapy. The 30-day mortality did not differ between ≥70 years and ≤69 years of age. The SCARF closely reflected the increasing risk of death in patients with increasing frailty (Fig. 2C) (OR for mild frailty 1.17 95% CI 1.07–1.28 as compared to severe frailty OR 1.48 95% CI 1.28–1.71). Although the risk of death in this group did not differ between ≥70 years and those ≤69 years of age, the overall mortality was much higher despite no clear differences in the number of patients with high SCARF index or poor ECOG PS.

Hospital admissions statistics during chemotherapy for lung cancer showed that admissions for patients ≥70 years old did not differ from the younger patients, and the OR for at least one admission within 6 months of the first day of systemic chemotherapy treatment was 0.78 for >70 y.o. (95% CI 0. 0.72–0.83). Patients who were frail, had a higher risk of admission than those who were not frail (OR for severe frailty 1.13; 95% CI 0.98–1.30). Similarly, poor ECOG PS was linked with a higher risk of admission (OR for ECOG PS 3 + 1.39; 95% CI 1.04–1.87).

## Discussion

The Primary Objective of this research was to define the relationship between SCARF and the adverse outcomes of chemotherapy in frail patients with cancer. The primary objective has been met, and the research shows that the SCARF index can be a useful indicator of 30-day mortality in frail patients with colorectal, breast and lung cancer.

We also showed that SCARF index does not predict hospital admissions related to chemotherapy toxicity. We show that patients with colorectal cancer and breast cancer treated with adjuvant chemotherapy, who are ≥70 years of age have a higher risk of dying than the younger patients.

For patients who are ≥70 years of age with lung cancer, SCARF index alone is not a good indicator of adverse outcomes with chemotherapy, and the frail cohort of patients does not have worse survival than the younger patients, however the mortality is much higher overall, in both groups. Patients with stage III and IV have cancer-related symptoms and the long-standing conditions that are described in SCARF index, bear less significance in comparison to the symptoms related to cancer. ECOG PS performs well overall; however, it is less precise and does not take into account pre-existent conditions. Although the large number of patients removes the investigator’s bias, the study has several limitations. It presents retrospective data and the accurate comparison between SCARF index and ECOG PS is not possible as PS is frequently not documented. It is also not possible to ascertain whether all admissions that occurred during chemotherapy were related to chemotherapy toxicity.

The difference in predictive power of SCARF index when comparing patients treated with adjuvant chemotherapy (breast and colorectal) with patients who have a large cancer burden (lung) indicates that additional assessments are needed. SCARF index takes into account chronic conditions that were present prior to cancer diagnosis. When faced with cancer-related symptoms such as pain, gastrointestinal symptoms or breathing difficulties related to underlying malignancy, it is clear that additional assessments of current health status are required.

The current online predictive tools such as CARG Score (Cancer and Ageing Research Group Chemotherapy Toxicity Risk Score) and CRASH (Chemotherapy Risk Assessment Scale for High-Age Patients) are valuable tools as they incorporate elements of geriatric assessment. This is currently recommended as a standard assessment by ASCO guidelines for Geriatric Oncology [22]. SCARF index does not include physical assessment, but it can be automatically calculated and is easy to obtain from medical health records. In the era of Artificial Intelligence, it can add valuable information as it appears to be an accurate indicator of frailty in patients with cancer. It does, however, differ from the originally described eFI [13]. Clegg et al. used electronic records derived from primary care and collected over a long time, whereas HES- derived clinical records rely on this information being entered onto the database during hospital admissions, therefore there may be differences between the two datasets [13]. SCARF index constructed from HES records, as opposed eFI based on GP records potentially introduces an element of under-ascertainment of medical conditions that are inadequately documented in HES; it is impossible to fully appreciate the magnitude of comorbidities that are missed in the SCARF index as calculated by HES.

From the statistical perspective, PS appears to be more predictive of adverse outcomes than the SCARF index. In clinical practice however, most clinicians agree that PS is an inadequate measure of frailty as it does not take into account the full picture of health. It can be altered by a simple short-lasting medical event, such as an acute infection and improve within hours of recovery. When using treatment such as chemotherapy, that can have lasting adverse outcomes, the assessment of pre-existing conditions is essential. PS alone is inadequate not only because it has a limited scope of enquiry but because, remarkably, it is often not recorded, even in a context where there is a focus on the comorbidities and frailty issues. In addition, since the COVID pandemic many pre-chemo assessments are done remotely via tele-consultations, making the PS estimate even less reliable. SCARF index adds value to the assessment of this group of patients even though the traditional and often neglected measure of PS is still making a contribution.

Going forward the electronic evaluation of patient frailty, such as SCARF index or GP-records derived eFI can be built into cancer patient evaluation at the point of referral and consultations and has the potential to lead to improved awareness of the key issues to be considered when taking shared decisions about major cancer interventions, for both patients and professionals. Obtaining the SCARF index does not require additional clinic time as it is already routinely available from electronic health records. The SCARF index in conjunction with PS could support clinical assessment and add to the discussion of the individual risks of chemotherapy as outlined by the above study and the fully informed decision can then be undertaken. Patients with frailty as assessed by SCARF would require a more in-depth geriatric assessment and could be referred to specialised geriatric teams to optimise their physical condition. Further research is required on how best to combine frailty index with assessment of cancer-related symptoms that affect physical health.

## Conclusion

The SCARF index predicts poor outcomes from SACT, particularly in early breast and colon cancer. Whilst geriatric assessment is the gold standard recommended by ASCO guidelines for Geriatric Oncology, SCARF can be a useful addition and requires further evaluation in a prospective study [22].

## Data Availability

Data can be obtained from: https://digital.nhs.uk/ndrs. https://digital.nhs.uk/ndrs/data/data-sets/sact. https://digital.nhs.uk/services/hospital-episode-statistics.
